# High-density Mapping Guided Pulmonary Vein Isolation for Treatment of Atrial Fibrillation - Two-year clinical outcome of a single center experience

**DOI:** 10.1038/s41598-019-45115-0

**Published:** 2019-06-20

**Authors:** J. Siebermair, B. Neumann, F. Risch, L. Riesinger, N. Vonderlin, M. Koehler, K. Lackermaier, S. Fichtner, K. Rizas, S. M. Sattler, M. F. Sinner, S. Kääb, H. L. Estner, R. Wakili

**Affiliations:** 1 Department of Cardiology and Vascular Medicine, West-German Heart and Vascular Center, University Duisburg-Essen, Essen, Germany; 2 Department of Medicine I, University Hospital Munich, Ludwig-Maximilians University, Munich, Germany; 3 German Cardiovascular Research Center (DZHK), partner site: Munich Heart Alliance, Munich, Germany; 4 Department of Cardiology, Heart Centre, Copenhagen University Hospital, Rigshospitalet, Copenhagen, Denmark

**Keywords:** Cardiology, Atrial fibrillation

## Abstract

Pulmonary vein isolation (PVI) as interventional treatment for atrial fibrillation (AF) aims to eliminate arrhythmogenic triggers from the PVs. Improved signal detection facilitating a more robust electrical isolation might be associated with a better outcome. This retrospective cohort study compared PVI procedures using a novel high-density mapping system (HDM) with improved signal detection vs. age- and sex-matched PVIs using a conventional 3D mapping system (COM). Endpoints comprised freedom from AF and procedural parameters. In total, 108 patients (mean age 63.9 ± 11.2 years, 56.5% male, 50.9% paroxysmal AF) were included (n = 54 patients/group). Our analysis revealed that HDM was not superior regarding freedom from AF (mean follow-up of 494.7 ± 26.2 days), with one- and two-year AF recurrence rates of 38.9%/46.5% (HDM) and 38.9%/42.2% (COM), respectively. HDM was associated with reduction in fluoroscopy times (18.8 ± 10.6 vs. 29.8 ± 13.4 min; p < 0.01) and total radiation dose (866.0 ± 1003.3 vs. 1731.2 ± 1978.4 cGy; p < 0.01) compared to the COM group. HDM was equivalent but not superior to COM with respect to clinical outcome after PVI and resulted in reduced fluoroscopy time and radiation exposure. These results suggest that HDM-guided PVI is effective and safe for AF ablation. Potential benefits in comparison to conventional mapping systems, e.g. arrhythmia recurrence rates, have to be addressed in randomized trials.

## Introduction

Pulmonary vein isolation (PVI) is an established therapy of atrial fibrillation (AF) aiming to eliminate ectopic triggers from the pulmonary veins (PV) which are suggested to play an important role in the initiation of AF^1,2^. It is suggested that complete isolation is required for successful ablation outcome^3^. Therefore, the procedural endpoint is an electrical isolation of the PVs following circumferential PV ablation. 3D-mapping systems with multipolar diagnostic catheters, used to test entry- and exit block to confirm complete electrical PVI, have been introduced in order to reduce radiation dose^4,5^, procedure duration^4–6^, and especially to improve outcome (recurrence) rates^7^. The most commonly used mapping catheter for assessment of electrical isolation in radiofrequency (RF) ablation is a circumferential diagnostic mapping catheter with 8–20 electrodes with an interelectrode distance of 2–6 mm^8^. It may be hypothesized that a higher number of and a smaller distance between electrodes (near-field signals) could result in better isolation by providing an increased resolution, which may translate in a better outcome. The novel high-density 3D-mapping (HDM) system (Rhythmia) provides high-resolution mapping using a 64-pole multipolar basket catheter (MBC) instead of the established circular mapping catheter of a conventional mapping system (COM)^9,10^. Recent data already suggested higher sensitivity for persistent atrio-PV conduction by HDM compared to COM, but outcome data is still missing^9^.

We hypothesized that PVI guided by HDM using the MBC for confirmation of PVI would allow for more accurate signal identification and therefore for a potentially more robust electrical PVI resulting in an improved clinical outcome compared to a COM-guided ablation approach.

## Methods

### Study population

We conducted a retrospective cohort study at the Department of Medicine I, University Hospital Munich, Ludwig-Maximilians University, Munich, Germany. We consecutively enrolled 54 patients with AF for first circumferential PVI between 04/2014 and 10/2016 with the HDM system. In addition, n = 54 patients, matched for age, sex and type of AF, were selected from a historic cohort (05/2009-03/2014) serving as controls, with the PVI performed by use of a conventional 3D-mapping system and a conventional circular 20-pole mapping catheter. Baseline patient demographics, clinical characteristics and outcome data were extracted from the patients’ hospital records. Procedural data for HDM-guided ablations were obtained during the ablation procedure, for COM-guided procedures from a self-designed AF database. The methods were carried out in accordance with the relevant guidelines and regulations. The protocol was approved by the institutional review board (IRB) and local ethics committee (Ethics Committee LMU Munich, protocol number: 494–16). All patients gave written informed consent prior to ablation.

### Rhythmia high-density mapping system and Orion MBC

The components of the HDM system have been described previously^9,11^. In brief, the Rhythmia system (Rhythmia, Boston Scientific, Cambridge, MA, USA) is a 3D-electroanatomical mapping system that uses hybrid location technology with impedance and magnetic localization^12^. By use of a magnetic field provided by a generator under the catheter table specific catheters capable of magnetic tracking can be visualized with an accuracy of ≤1 mm^12^. Impedance measurement is mainly needed for navigation of catheters within the 3D shell which are not capable of magnetic spatial localization. A MBC (IntellaMap Orion, Boston Scientific, Marlborough, MA, USA; 1.8 cm diameter, 64 electrodes; 2.5 mm inter-electrode spacing center-to-center) enables high resolution mapping without the need of manual annotation. The mapping catheter can be used for mapping un-deployed (3 mm diameter) to a fully deployed condition (22 mm diameter)^13,14^. For geometry assessment, the 64 bipolar electrodes are used to acquire points with every accepted beat. For intubation of small structures, like PVs and left atrial (LA) appendage, the basket catheter is inserted in the vein/appendage and then gently deployed until wall contact is established.

### Procedural study protocol

Pre-procedurally, patients on non–vitamin K anticoagulants (NOACs) were encouraged to pause this medication on the morning of the procedure day and continued the treatment after the PVI. In case of a vitamin K anticoagulant (VKA) treatment prior to the procedure, patients continued this medication throughout the procedure aiming for an international normalized ratio of 2.0–2.5 on the day of the procedure.

In both study groups, a transesophageal echocardiography was performed the day before PVI or peri-procedurally to rule out thrombi in LA/LA appendage. Procedures were performed by three experienced operators applying conscious sedation by use of intravenous midazolam and remifentanil. Radial artery access was obtained to place a pigtail catheter into the posterior cusp of the aortic valve for anterior/posterior orientation during transseptal puncture and for continuous invasive hemodynamic monitoring. Catheter access was performed by 6, 7 and 9 French sheaths inserted in the right femoral vein. A decapolar diagnostic catheter was placed in the coronary sinus for mapping reference and orientation during transseptal puncture. Transseptal puncture was performed using a single steerable sheath (Agilis^TM^ Steerable Introducer, St. Jude Medical, St. Paul, MN, USA) by fluoroscopic and pressure guidance. After accessing the LA, periprocedural anticoagulation was started with unfractionated heparin. Thereafter, PV angiography was performed by use of a 6 F diagnostic catheter. The ablation catheter was introduced into LA passively through the same transseptal orifice without a second transseptal sheath. Once the ablation catheter was placed safely in the LA, the 3D mapping system was used in order to generate LA geometry by HDM or COM, respectively.

### 3D Mapping

In the HDM group, the 3D electro-anatomical shell of the LA and the PVs including the LA appendage were obtained using the HDM system. Specific map acquisition details are described elsewhere^14^. In brief, the MBC was maneuvered gently inside the LA structures with automatic acquisition of both voltage points and geometry without manual annotation according to established criteria including stable cycle length and catheter location stability^15^.

In the COM group, mapping of the LA and the PVs was performed using the CARTO-3 system (Biosense Webster, Inc., Baldwin Park, CA, USA). Instead of the MBC, a conventional diagnostic circular mapping catheter was used for geometry assessment and voltage mapping. After 3D mapping, the diagnostic MBC or circular catheter remained in the LA to monitor the acute efficacy of RF delivery on PVI.

### Ablation procedure

All ablations were performed by use of a standard single-tip catheter. In the HDM group, ablations were performed using an open-irrigated single tip catheter (3.5 mm irrigated tip, Biosense Surround Flow, Biosense Webster, Diamond Bar, CA, USA). Ablations in the COM group were performed using a Surround Flow or a SmartTouch catheter (both Biosense Webster, Inc., Baldwin Park, CA, USA, 3.5 mm open-irrigated tip), by a point-by-point ablation approach. Maximum delivered RF energy for both groups did not exceed 30 watts, delivered by a 500 kHz ablation unit (Stockert EP shuttle, Biosense Webster, Inc., Baldwin Park, CA, USA). Each RF delivery was limited to 240 seconds. Impedance, temperature and power were continuously monitored and were visible to the electrophysiologist through the whole procedure.

### Periprocedural anticoagulation

Periprocedural anticoagulation was performed with unfractionated heparin intravenously, aiming for an activated clotting time of 300–350 seconds. A heparin bolus application (80–100 international units/kg) was followed by continuous intravenous administration throughout the procedure duration and activated clotting time was assessed at least every 30 minutes.

### Testing for complete electrical PVI

If patients did not convert spontaneously from AF to sinus rhythm during RF delivery, electrical cardioversion was performed after circumferential isolation to confirm complete PVI by entrance and exit block testing. In the HDM group the MBC, in the COM group a regular 20-pole circular mapping catheter was used to verify complete electrical conduction block from the atrium into the PVs (entrance block). The definition of entrance block was complete absence of PV potentials adjudicated by the operator. PV potentials were suspected when signals were high-frequency electrograms with a sharp upstroke, duration <50 ms, and amplitude >0.05 mV. Atrial far-field electrograms were defined as low-amplitude, low-frequency signals and were in doubt confirmed by differential pacing from the LAA or the free wall of the LA. Exit block testing was performed with the MBC at least with all equatorial electrodes (HDM group) or the circular mapping catheter (COM group) from at least 3 separated bi-poles showing the non-excitability of atrial myocardium by pacing from the PVs due to complete PVI^1^.

### Clinical follow-up

According to local clinical routine, follow-up was performed at least at 6 and 12 months after PVI, and additionally in 6 month intervals if applicable. This follow-up included physical examination, evaluation of symptoms indicating AF recurrence, 12-lead electrocardiogram (ECG) recordings and a 7-day Holter ECG. Recurrence of AF was defined as at least one documented episode >30 s of AF on a Holter ECG recording or AF documented on a 12-lead ECG. Occurrence of atypical atrial flutter or atrial tachycardia was considered equivalently as AF recurrence for the analysis of the primary efficacy endpoint. Palpitations without ECG documentation of AF were not considered AF recurrence. Antiarrhythmic drugs were discontinued after the 90 days blanking period.

### Study endpoints

The primary efficacy endpoint was freedom from any symptomatic atrial arrhythmia recurrence. A subanalysis aimed at the identification of potential clinical covariates associated with the primary endpoint using an uni- and multivariate analysis. The procedural endpoint was complete electrical isolation of all PVs, confirmed by the MBC in HDM-guided procedures or the circular mapping catheter in COM-guided procedures. Secondary endpoints were total procedure time (including preparation time) and LA mapping duration, fluoroscopy time, and radiation dose. Procedure time including preparation time was defined as the entire time span the patient was in the catheter lab. Safety endpoints comprised death, pericardial effusion/tamponade, bleeding BARC ≥2, phrenic nerve palsy, cerebrovascular events (transitory ischemic attack/stroke), or significant hematoma at the access site.

### Statistical analyses

Data are presented as mean ± standard deviation (SD) unless otherwise indicated. After testing for normality (Levene - Test) an unpaired Student’s t-test was used for two-group comparisons of continuous variables. For categorical variables, analysis was performed by chi-square or Fisher’s exact tests as applicable. We plotted survival curves using the Kaplan–Meier estimation and compared differences by log-rank tests, censored to 730 days.

Univariate assessment for a potential association with AF recurrence was performed for the baseline patient characteristics. For the multivariate model an adjustment for univariate covariates with an empirically chosen threshold of a hazard ratio for AF recurrence below 0.80 and >1.30 was performed. A two-tailed p-value of 0.05 was considered significant. Analyses were performed using SPSS (version 20.0; SPSS Institute, Chicago, IL, USA).

## Results

### Patient characteristics

Patient characteristics are listed in Table 1. Patients in the HDM and the COM group had a comparable age of 65.0 and 62.9 years (p = 0.32), respectively. No significant differences were noted for sex, left ventricular ejection fraction, LA diameter, CHA_2_DS_2_-VASc score, and cardiovascular risk factors. Also, percentages of patients with paroxysmal AF were comparable between the two study groups (HDM group 53,7% and COM group 48.1%, p = 0.56).

**Table 1:** Baseline patient characteristics.

	Overall (n = 108)	HDM group (n = 54)	COM group (n = 54)	p-value
Paroxysmal AF, n (%)	55 (50.9)	29 (53.7)	26 (48.1)	0.56
Age, years	63.9 ± 11.2	65.0 ± 12.0	62.9 ± 10.3	0.32
Male sex, n (%)	61 (56.5)	28 (51.9)	33 (61.1)	0.44
CHA^2^DS^2^-VASc	2.5 ± 1.6	2.6 ± 1.8	2.3 ± 1.5	0.41
LVEF, %	61.5 ± 10.9	59.9 ± 9.7	62.9 ± 11.7	0.17
LA diameter, mm	40.9 ± 7.1	40.3 ± 7.7	41.3 ± 6.7	0.51
Smoking, n (%)	21 (19.4)	11 (20.4)	10 (18.6)	0.96
Diabetes mellitus, n (%)	15 (13.9)	8 (14.8)	7 (13.0)	0.91
Stroke, n (%)	13 (12.0)	7 (13.0)	6 (11.1)	0.85
Hypertension, n (%)	77 (71.3)	38 (70.4)	43 (79.6)	0.38
Coronary artery disease, n (%)	22 (20.4)	13 (24.1)	9 (16.7)	0.42
Betablocker, n (%)	95 (88.0)	46 (85.2)	49 (90.7)	0.52
Class I AAD, n (%)	19 (17.6)	10 (18.5)	9 (16.7)	0.80
Class III AAD, n (%)	16 (14.8)	10 (18.5)	6 (11.1)	0.28

HDM, high density mapping; COM, conventional mapping; AF, atrial fibrillation; LVEF, left ventricular ejection fraction; LA, left atrial; AAD, anti-arrhythmic drugs.

### Procedural and efficacy endpoints

**Figure 1:**
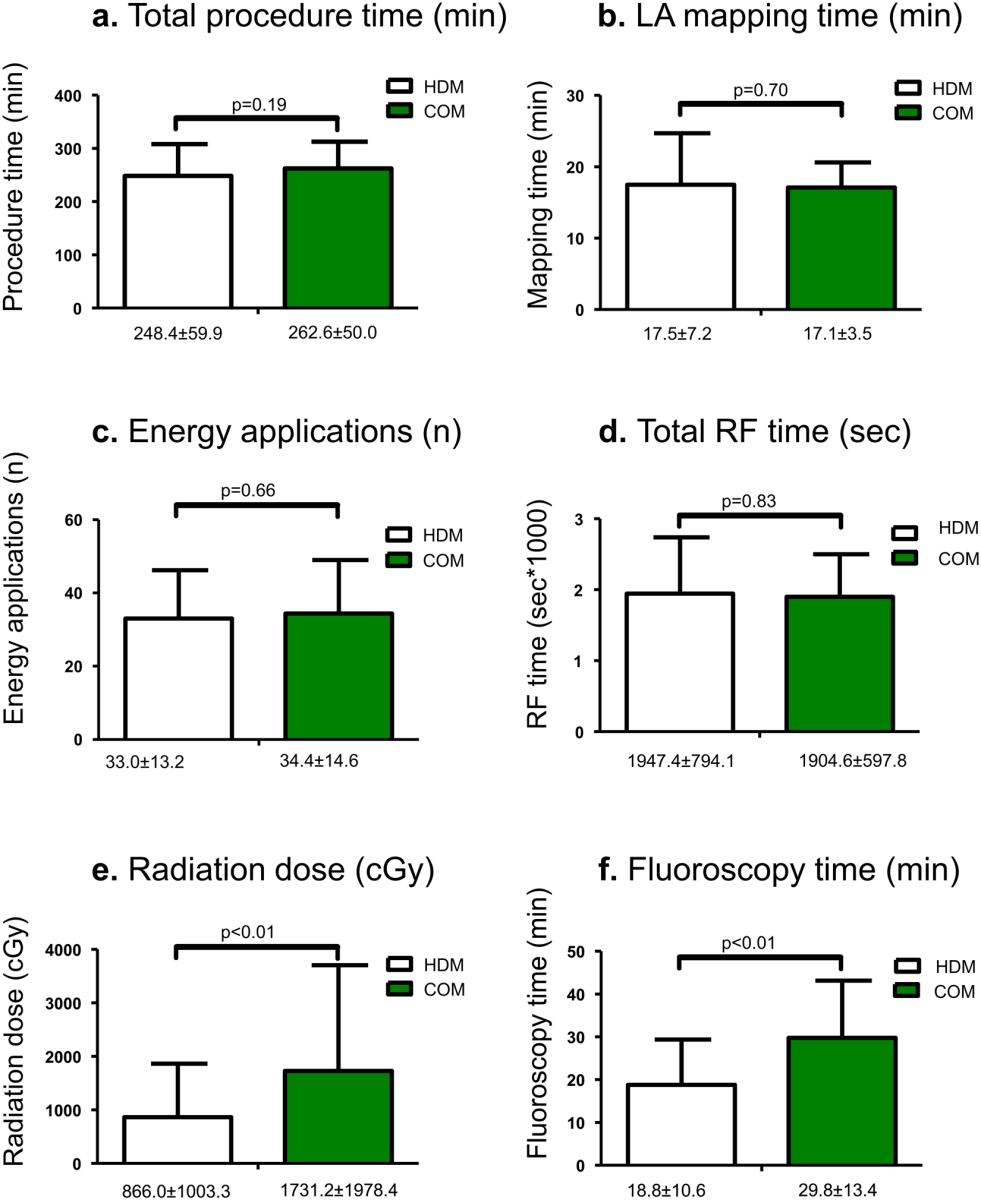
Graphical illustration of procedural parameters. Total procedure time (**a**), total LA mapping time (**b**), number of radiofrequency energy applications (**c**), and total radiofrequency time (**d**) were not significantly different between groups. Compared to the conventional 3D mapping group (COM), radiation dose (**e**) and total fluoroscopy time (**f**) were significantly lower in the high density mapping group (HDM).

Complete electrical PVI was achieved in all patients in both groups. Figure 1 depicts the periprocedural parameters between patients undergoing ablation by the HDM- and the COM-guided procedures. Fluoroscopy times (18.8 ± 10.6 vs. 29.8 ± 13.4 min, p < 0.01) and total radiation dose (866.0 ± 1003.3 vs. 1731.2 ± 1978.4 cGy, p < 0.01) were significantly lower in the HDM cohort. However, total procedure time (248.4 ± 59.9 vs. 262.6 ± 50.0 min) and LA mapping time (17.5 ± 7.2 vs. 17.1 ± 3.5 min) were not different between the HDM and the COM groups. The number of radiofrequency energy applications as well as the cumulative radiofrequency time required for complete electrical isolation were comparable between the HDM and the COM group.

### Clinical outcomes

**Figure 2:**
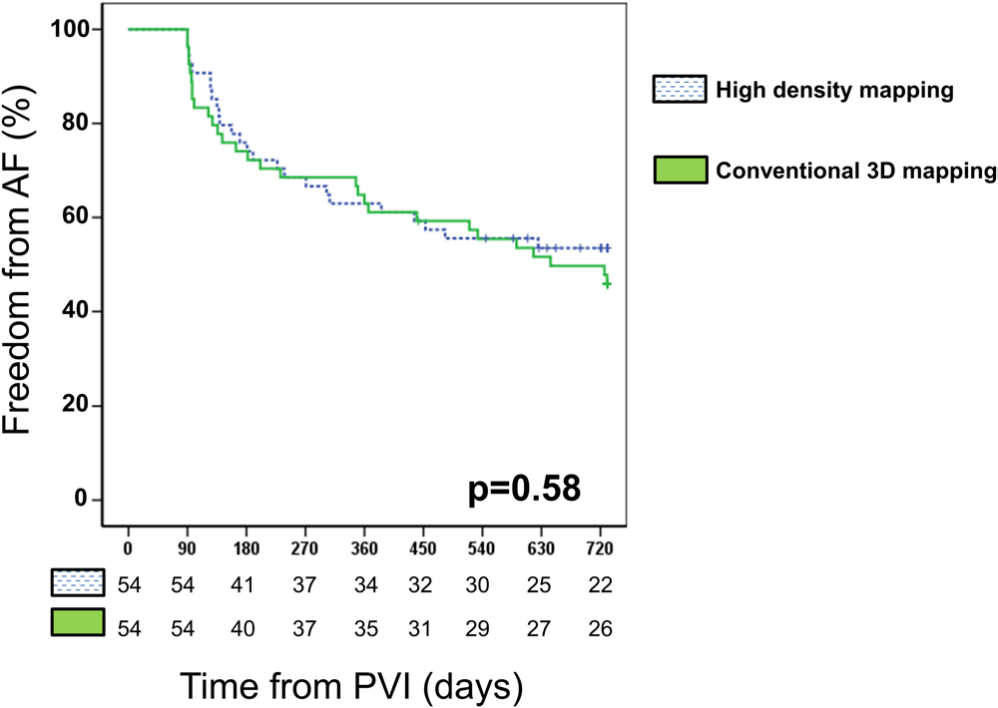
Mid-term AF recurrence rates after first PVI. Kaplan-Meier Kaplan-Meier curve illustrate the clinical outcome of AF recurrence during follow-up. No significant difference was observed between the High-Density mapping (HDM) guided and the conventional 3D-mapping (COM) guided procedures over the follow-up period.

The mean FU duration was 494.7 ± 26.2 days. All patients were off antiarrhythmic drugs after the 90-days blanking period. With respect to arrhythmia recurrence, clinical mid-term outcome was similar in the two studied groups (hazard ratio [CI] 1.16 [0.68; 1.99], p = 0.58). The probability of AF recurrence in both groups is illustrated in Fig. 2. One- and two-year recurrence rates of any atrial arrhythmia were 38.9% and 46.5% in the HDM group and 38.9% and 42.2% in the COM group, respectively. Looking at the subgroup of patients with paroxysmal AF there was no significant difference with respect to one- and two-year recurrence rates (HDM group: 27.6%/31.4% vs. COM group: 42.3%/46.2%; p = 0.24). The same was the case for the subgroup of patients with persistent AF showing no differences in one- and two-year recurrence rates (HDM group: 48%/64% vs. COM group: 32.1%/58.4%; p = 0.42).

The univariate analysis for other relevant clinical covariates failed to identify any variable associated with the primary endpoint (Table 2). Adjusted for covariates with a hazard ratio for AF recurrence below <0.80 and >1.30 (paroxysmal AF, male sex, diabetes mellitus, stroke, hypertension, and coronary artery disease), the use of HDM could not be shown to be associated with the endpoint of symptomatic atrial arrhythmia recurrence (HR 1.15 [0.66; 2.00], p = 0.63).

**Table 2:** Univariate assessment of covariates associated with the primary endpoint.

	Univariate analysis	
	Hazard ratio	p-value
Paroxysmal AF, n (%)*	0.60 [0.35; 1.04]	0.07
Age, years	1.02 [0.99; 1.04]	0.30
Male sex, n (%)*	0.79 [0.46; 1.34]	0.79
CHA2DS2-VASc	1.04 [0.87; 1.24]	0.68
LVEF, %	0.99 [0.97; 1.02]	0.45
LA diameter, mm	1.02 [0.98; 1.06]	0.36
Smoking, n (%)	1.08 [0.56; 2.11]	0.81
Diabetes mellitus, n (%)*	0.75 [0.32; 1.76]	0.51
Stroke, n (%)*	2.08 [1.01; 4.27]	0.06
Hypertension, n (%)*	1.43 [0.74; 2.78]	0.29
Coronary artery disease, n (%)*	1.38 [0.74; 2.57]	0.32
Betablocker, n (%)	1.23 [0.49; 3.09]	0.66
Class I AAD, n (%)	1.26 [0.65; 2.43]	0.50
Class III AAD, n (%)	1.10 [0.53; 2.23]	0.82

The univariate variables with an empirically chosen threshold of a hazard ratio below <0.80 and >1.30 for the primary endpoint of any symptomatic atrial tachycardia (highlighted with an asterisk) were used for the multivariate model. AF, atrial fibrillation; LVEF, left ventricular ejection fraction; LA, left atrial; AAD, antiarrhythmic drugs.

### Safety endpoint

No cerebrovascular events or pericardial effusion/tamponade were observed. In the HDM group, two cases of hematoma at the venous access site in the femoral vein without the need for transfusion occurred. In the COM group, one case of significant groin hematoma occurred. None of the vascular complications required interventional or surgical treatment.

## Discussion

The main finding of this cohort study was that PVI guided by a HDM system with a 64-pole MBC was equivalent to ablation guided by a COM system using an 8-polar circular mapping catheter with respect to freedom from AF during mid-term follow-up in a representative PVI patient cohort with evenly distributed percentages of paroxysmal and persistent AF.

### Pulmonary vein mapping

Based on our present data there was no difference in outcome over 2 years of follow up. The main question arising is if there is actual a difference in the accuracy of electrical isolation by using this novel type of MBC. Available literature suggests that higher electrode density enables detection of small signals allowing the identification of residual PV conduction not capable for regular mapping catheters with either lower electrode numbers, larger electrode size or spacing^9,16–18^. A recent study investigated this specific issue comparing a circular mapping catheter to HDM mapping (Rhythmia system) using a MBC simultaneously in the same PV with respect to signal concordance before and after PVI^9^. The main result was an overall good concordance of recordings before ablation. Following circular radiofrequency PVI, the concordance between the catheter signals decreased to 68%, mainly due to persistence of PV potentials on the MBC, which were not visualized on the circular mapping catheter. This led to an overestimation of complete PVI when using only a circular mapping catheter with the potential for a higher AF recurrence rate. The authors speculated that the smaller and closer space between the electrodes and the higher number of bi-poles (64 vs. 20 electrodes on the MBC and on the circular mapping catheter, respectively) to assess ablated tissue provided better accuracy in gap identification. Further, they indicated that smaller electrodes with smaller interelectrode spacing were less subject to signal averaging and cancellation effects, with finally providing electrograms with higher bipolar voltage amplitude^9^. Another study showed that in cryoballoon-based PVI procedures a high density, 32-pole multipolar catheter was superior in identifying PV potentials before and after PVI compared to a standard octapolar spiral catheter^18^.

Nevertheless, our study failed to show superiority of the HDM-guided approach with respect to clinical outcome despite a possibly more robust electrical PVI.

### Role of electrical PV isolation

Considering our result that better electrical isolation in HDM- vs. COM-guided procedures did not translate into better outcomes the question arises if there is always the need for a complete electrical PVI to optimize success rates after PVI. This is relevant especially in the context that our study enrolled patients with paroxysmal and persistent AF for catheter ablation therapy.

Since the group of Haissaguerre described that ectopic foci from the PVs could be triggers of AF, most ablation approaches aimed at complete electrical isolation of the atria from the PVs^2^. In patients with AF recurrence several studies reported a high incidence of PV reconnection after ablation contributing to the hypothesis of PV ectopy triggering AF^19,20^. Since multipolar mapping catheters are a crucial tool for assessing PV isolation, the accuracy of signal detection by this catheter and complete elimination of PV potentials have been suggested to be a critical part of the procedural endpoint of a catheter-based AF treatment. However, studies for direct comparison of distinct PV mapping guided ablation approaches on outcome are scarce. Meissner *et al*. compared 24 patients with PVI confirmation by a multipolar 32-pole catheter with 167 patients investigated with a regular circular mapping catheter for signal verification. The authors found no significant differences in short 3 and 6 months outcomes^18^. In another study of this group, the authors did also report on comparable success rates with respect to short- and mid-term outcomes after PVI irrespective of the use of an a multipolar 32-pole diagnostic catheter^21^. Those findings are in line with our results comparing HDM vs. COM-guided PVI. Yet, all available studies are limited by low patient numbers. PV reconnection can be identified in most patients with AF recurrence undergoing re-do procedures suggesting at least an association of re-connection on recurrence. However, there is only limited data investigating PV reconnection in patients without AF recurrence^19,20,22^. The GAP-AF and Pressure trials constitute a few examples which challenged the concept of complete isolation^3,23^. The GAP-AF trial suggested superiority of a complete over incomplete PVI regarding short-term outcome rate and was one of few trials invasively investigating all patients after three months irrespective of AF recurrence to evaluate PV reconnection^3^. Consistent with the outcome, the PV reconnection rate comparing both strategies was higher in the incomplete PVI study group. However, within each strategy arm the reconnection rate did not differ between patients with and without AF recurrence, suggesting a more relevant role of the strategy itself rather than a durable complete electrical PVI. The latter would also be in line with our data since we have performed a similar ablation strategy in all patients.

There are a few other studies supporting this theory; one study re-investigated successfully treated patients without AF recurrence and observed that 90% of these patients showed at least one PV reconnected despite stable sinus rhythm after PVI^24^. Furthermore, re-ablation studies in heart failure patients revealed that AF recurrence also occurs despite persistence of completely isolated veins^25^.

Hence, further research has to be performed in order to evaluate in which patients PV isolation might be more or less important to facilitate a benefit by a HDM guided ablation approach in a specific group of patients.

### Non-PV related AF mechanisms and ablation targets

Some non-PV related ablation concepts are emerging^26^, including a potential role of the so-called atrial rotor ablation^27^. Whereas the DECAAF-I trial provided evidence for fibrosis as a significant determinant of PVI ablation success^28^, the DECAAF-II trial^29^ is evaluating the concept of a MRI-guided fibrosis-targeted ablation in patients with persistent AF. It is still challenging to elucidate the relative contribution of an arrhythmogenic substrate vs. PV triggers to AF in the individual patient. In our study we included around half of all patients with persistent AF, in which some of the suggested substrate-based mechanisms might play a role beyond the PV triggers.

### Role of HD mapping in AF ablation procedures

Our study showed that total procedure time and LA mapping time did not differ between the two groups, indicating that HDM due to the auto-annotation of intra-cardiac signals did actually not translate into longer mapping or procedure times. However, radiation dose and total fluoroscopy time were significantly lower in the HDM group suggesting that the mapping by MBC might allow for a better visualization requiring less fluoroscopy. At this point, it is hard to draw a valid conclusion for a procedural benefit due to our non-randomized approach. Our results confirm earlier feasibility studies demonstrating comparable complication rates of a HDM-guided ablation approach. In terms of potential extra-PV ablation targets it can be speculated that a detailed high-resolution anatomical and electrical mapping by an MBC might offer opportunities for an improved treatment strategy in the future, e.g., low-voltage area ablation.

### Study limitations

We report outcome data on the use of a novel HDM system in patients undergoing PVI for AF treatment in a low number of patients. Due to consecutive patient enrolment the presented data do not represent randomized results. As with the introduction of the novel mapping system most procedures have been performed with the HDM system, patients from a historic cohort were chosen to serve as controls for a matched comparison. Nevertheless, all procedures have been performed by the same operators and according to an identical workflow. Further, ECG assessment was only performed at pre-defined time points or when symptoms occurred, no continuous ECG monitoring was performed. Therefore, some episodes of AF could be missed during the follow-up period and the assessment of the cumulative AF burden was not possible.

## Conclusion

A HDM-guided ablation approach for AF, presumably offering better signal quantity and quality, was equivalent but not superior to COM-guided PVI procedures with respect to freedom from AF in patients with paroxysmal and persistent AF. Our data suggest that HDM-guided PVI is safe and efficient with a potential benefit on fluoroscopy time and radiation dose reduction. Further research is warranted to evaluate the value of high-density mapping by MBC catheters in AF ablation procedures with respect to outcome and procedural parameters in larger pre-specified patient groups.

## Data Availability

The datasets generated during and/or analyzed during the current study are available from the corresponding author on reasonable request.
